# Dataset of manual metal arc welded heterogeneous thin steel plates AISI1018 AND AISI4340

**DOI:** 10.1016/j.dib.2023.109572

**Published:** 2023-09-14

**Authors:** Titus Wanazusi, Milon Selvam Dennison, Stephen Ndubuisi Nnamchi

**Affiliations:** School of Engineering and Applied Sciences, Kampala International University, Western Campus, Uganda

**Keywords:** Manual metal arc welding, Heterogeneous welding, Welding factors, Tensile strength, Full factorial design, Analysis of variance, Regression analysis

## Abstract

Advancement in technology demands the joining of heterogeneous metals of low and high-carbon steel grades. In this investigation, AISI1018 low and AIS4340 medium carbon steels were welded to form a heterogeneous thin metal joint using the Manual Metal Arc Welding (MMAW) method. Experimental variations of welding current, electrode position, and weld orientation are selected as the MMAW parameters. The trials are planned using the Full Factorial Design (FFD) and the trial results are analysed using Analysis of Variance (ANOVA) and regression methods. A computerized tensile testing machine (TM2101N) was used to test the tensile strength of the welded specimens that were prepared in accordance with the ASTM E646 – 98 standards. The prediction model for tensile strength was generated based on regression analysis. The ANOVA and prediction model helped in studying the effect of the MMAW parameters.

Specification Table

**Table:** 

Subject	Industrial and Manufacturing Engineering

Specific Subject	Welding Technology
Data Format	Raw and analysed
Type of data	Table, Image, Chart, and Graph
How data were acquired	The data were acquired by conducting the trials.
Data collection	The data collection was performed by welding heterogeneous 2mm thin steel plates of AISI 1018 and AISI 4340 via Manual Metal Arc technique and its effect was investigated for the parameters such as welding current, weld orientation, and electrode position on tensile strength measured using a Universal Testing Machine (TM2101N). The supplementary responses weld bead geometry and weld deposition are measured using a digital vernier caliper and a precision weighing machine respectively.
Data Source Location	Mechanical Engineering Workshop, Department of Mechanical Engineering, Kampala International University, Western Campus, P.O. Box 71, Bushenyi-Ishaka, Uganda (0.5384° S, 30.1448° E)
Data Accessibility	Repository name: Mendeley Data Data identification number: 10.17632/j6657wk3tf.1 Direct URL to data: https://data.mendeley.com/datasets/j6657wk3tf/1

## 1 Value of the Data

• The dataset presented in this article conveys the feasibility of welding AISI1018 AND AISI4340 thin steel plates
• The dataset presented in this article gives the knowledge of selecting the optimum MMAW conditions for welding heterogeneous thin steel plates.
• The dataset presented in this article can be utilized to produce quality manual metal arc weldments.
• The dataset presented here can be used by researchers to compare the MMAW performance of heterogeneous thin steel plate welding.

## 2 Data Description

With the advancement in technology, always there will be a call for heterogeneous metal welding, especially in the engineering material steel alloys [1], [2], [3], [4]. The steel alloy AISI1018 is the most commonly used and cheap steel grade in the fabrication industry for making doors, window grills, and stair guards [5]. The failure rate through the material is high as it is easily affected by corrosion. The steel alloy AISI4340 is a medium carbon steel that offers high tensile strength and appreciable resistance to corrosion [6]. However, this material has poor weldability as associated with spatter and high slag formation [3,6]. The high spatter in welding AISI4340 yields porosity, incomplete fusion, and shallow penetration defects. Nowadays, for plenty of commercial applications, there is a need to join AISI1018 with AIS4340. This kind of heterogeneous welding requires a variety of manual metal arc welding currents, weld orientation, and position of the electrode for achieving the best weld quality. This research work started with identifying the engineering problems of dissimilar welding. After that, the work was stimulated with the selection of materials, and scientific methods were adapted such as material testing, selection of welding electrodes, planning the trials through Design of Experiments (DoE), heat treatment of electrodes, selection of fixtures, Arc welding machine (3 phase 220V/380V Dual Voltage IGBT 400/500/630A industrial Inverter MMA/stick Welder) and testing equipment such as Universal Testing Machine (TM2101N), weld bead geometry measured using a digital Vernier Caliper and weld deposition measured using a precision weighing machine. All the data observed in this project work has been sincerely recorded and stored in the data repository medium Mendeley Data (https://data.mendeley.com/datasets/j6657wk3tf/1).

## 3 Experimental Design, Materials and Methods

**Fig. 1:**
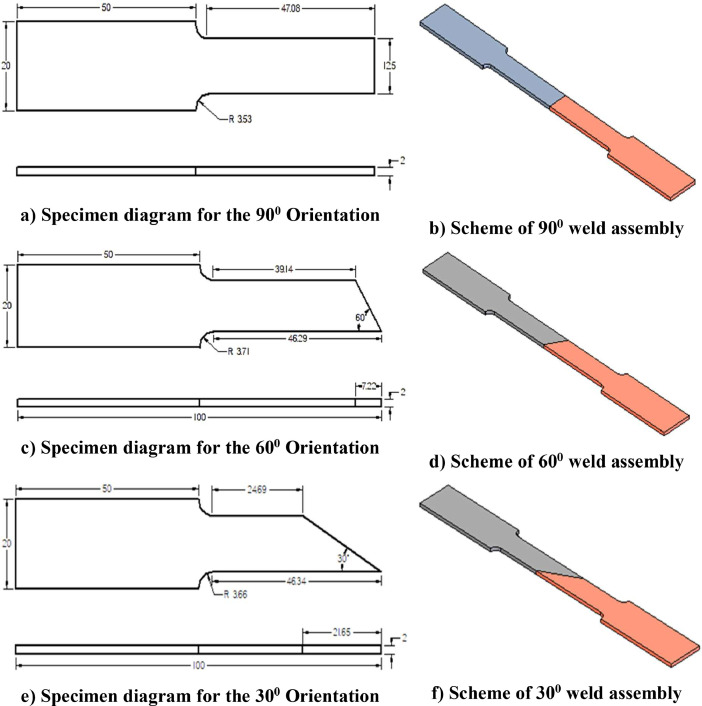
Scheme of Specimen Preparations.

This data article adapted the Full Factorial design (FFD) and regression analysis to study the effect of manual metal arc welding parameters such as welding current, weld orientation, and electrode position on tensile strength and also associated responses such as weld bead height, and weld deposition. The trials were planned with three levels of each input welding parameter with a replica of a set of 27 experimental runs. For this case, 54 workpieces of AISI1018 and 54 workpieces of AISI4340 were prepared in accordance with ASTM E646 – 98 for the trials. Since measurements were to be precise, a digital Vernier Caliper was employed to confirm these measurements with the thickness of the thin plate in this experiment. The scheme of the specimen preparations is portrayed in Fig. 1.

The mass data of welding specimens before and after the trials were recorded using a precision weighing machine, the tensile strength data of the weldments was tested and observed using a Universal Testing Machine (TM2101N) and finally the weld bead height was measured using a precise digital Vernier Caliper.

Workpiece preparation comprised cutting the workpieces to appropriate dimensions, weld angle, linear dimensions, and grinding off the sharp edges. A hand electric cutting machine was used for cutting the workpiece lengthwise as the hand saw was used for cutting along the width of the workpieces for precise dissection. A hand-electric grinding machine was used to clean off any excess flash and sharp edges from the specimens.

The welding electrode (E6013) was heat treated by baking at 110°C for 15 minutes in a muffle furnace to reduce weld spatter. This was essential in that medium carbon steel is difficult to weld as it yields a lot of spatter and other weld-related defects and elimination of absorbed moisture. In this way, the optimized weld condition of the dissimilar steel plates for the best weld joint was reported. Likewise, the prepared specimens were heat treated at 160°C and the temperature was held for 30 minutes which was necessary to eliminate moisture on the specimens before welding, and allowed to cool inside the furnace to room temperature.

A Manual metal arc welding machine (3 phase 220V/380V Dual Voltage IGBT 400/500/630A Industrial Inverter MMA/Stick welder) was used to join dissimilar steel sheets (AISI1018 and AISI4340). The joined specimens were then subjected to tensile testing using the TM2101N tensile testing machine. The parameters and the levels considered in this study are given in Table 1. Twenty-seven (27) experiments were developed using the Full Factorial Design (FFD) including one repetition aimed at minimizing noise during actual experiments. The outcome of the FFD-based experimental data such as tensile strength, weld deposition, and weld bead height are presented in Table 2.

**Table 1:** Welding Parameters and Levels.

Parameters	Notation	Level 1	Level 2	Level 3
Welding current (Ampere)	I	40	60	80
Electrode position (Degree)	E	30	60	90
Weld orientation (Degree)	w	30	60	90

**Table 2:** Experimental Data.

The results were analysed in Minitab Version 21.3 (64-bit) statistical software. The linear model was found to best fit the results, meanwhile, linear plus 2-way interaction was adopted for developing the regression model. Inbuilt ANOVA Minitab generated a coded and actual equation (regression model) that was used in predicting responses at any given set of input values as seen in Eq. (1) with R-squared (R^2^=97.47 %).

**Fig. 2:**
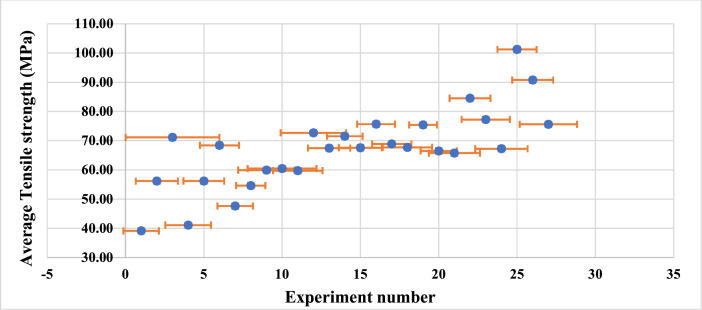
Data Error Bar Plot.

The error bars are a graphical representation of standard error, standard deviation, or percentage value informed by bars that indicate the relation in data variables. The bars are cap-tipped lines extending from the centre of the plotted data point by which their length displays the uncertainty of a data point. It helps to comprehend the variability of data given in two-dimensional structures, indicating the estimated error which gives the general understanding of the measured data points. The error bar plot in Fig. 2 portrays the variation between data points of tensile strength obtained with respect to two sets of 27 experimental runs in this study.

The statistical technique Analysis of Variance (ANOVA) is used to govern the difference significance between the groups [7]. This statistical analysis provides overall indicator data, such as the F-value, and its associated *p*-value [7]. If the *p*-value is below a predetermined significance level (0.05), it indicates that there is sufficient evidence to reject the null hypothesis and conclude that there are significant differences between the group means [8,9]. ANOVA allows for inferences about the effects of the selected MMAW parameters on the response output. It helps to determine whether the parameters have a significant impact on the response output. By assessing the F-value and associated *p*-value for each parameter, we can determine if the parameter has a significant effect on the response output data. The ANOVA analysis is given in Table 3 which gives the proposed model for Tensile strength.

(1) $$\begin{array}{ccc} T\left(\text{MPa}\right) & = & -\;5.85+21.66\;I\left(A\right)+2.80\;E\left(\text{degree}\right)+31.31\;w\left(\text{degree}\right)+5.340\;I\left(A\right)*E\left(\text{degree}\right)\\ & & -\;10.343\;I\left(A\right)\;*w\left(\text{degree}\right)-4.657\;E\left(\text{degree}\right)*w\left(\text{degree}\right) \end{array}$$

**Table 3:** Analysis of Variance.

Source	DF	Seq SS	Contribution	Adj SS	Adj MS	F-Value	*p*-value
Model	6	4674.68	97.47 %	4674.68	779.11	128.46	0.000
Linear	3	2788.62	58.14 %	1827.52	609.17	100.44	0.000
I (A)	1	2445.45	50.99 %	649.75	649.75	107.13	0.000
E (degree)	1	312.46	6.52 %	10.66	10.86	1.79	0.196
w (degree)	1	30.71	0.64 %	1356.95	1356.95	223.74	0.000
2-way interaction	3	1886.06	39.33 %	1886.06	628.69	103.66	0.000
I (A)*E (degree)	1	342.13	7.13 %	342.13	342.13	56.41	0.000
I (A)*w (degree)	1	1283.71	26.77 %	1283.71	1283.71	211.67	0.000
E (degree)*w (degree)	1	260.21	5.43 %	260.21	260.21	42.91	0.000
Error	20	121.30	2.53 %	121.31	6.06		
Total	26	4795.97	100.00 %				

In the statistical model of this research, the visual insights of the relationship and interaction between the MMAW parameters on the output response tensile strength can be well understood using surface plots [10]. It allows the researchers to analyze how changes in the selected MMAW parameters affect the output response and provide an intuitive way to interpret the model's behavior. The surface plots also aid in determining the direction of influence of each MMAW parameter on the response. By observing the shape of the surface, we can identify whether the response increases or decreases with changes in the parameters. Additionally, the steepness or flatness of the surface indicates the strength of the relationship between the MMAW parameters and the response.

**Fig. 3:**
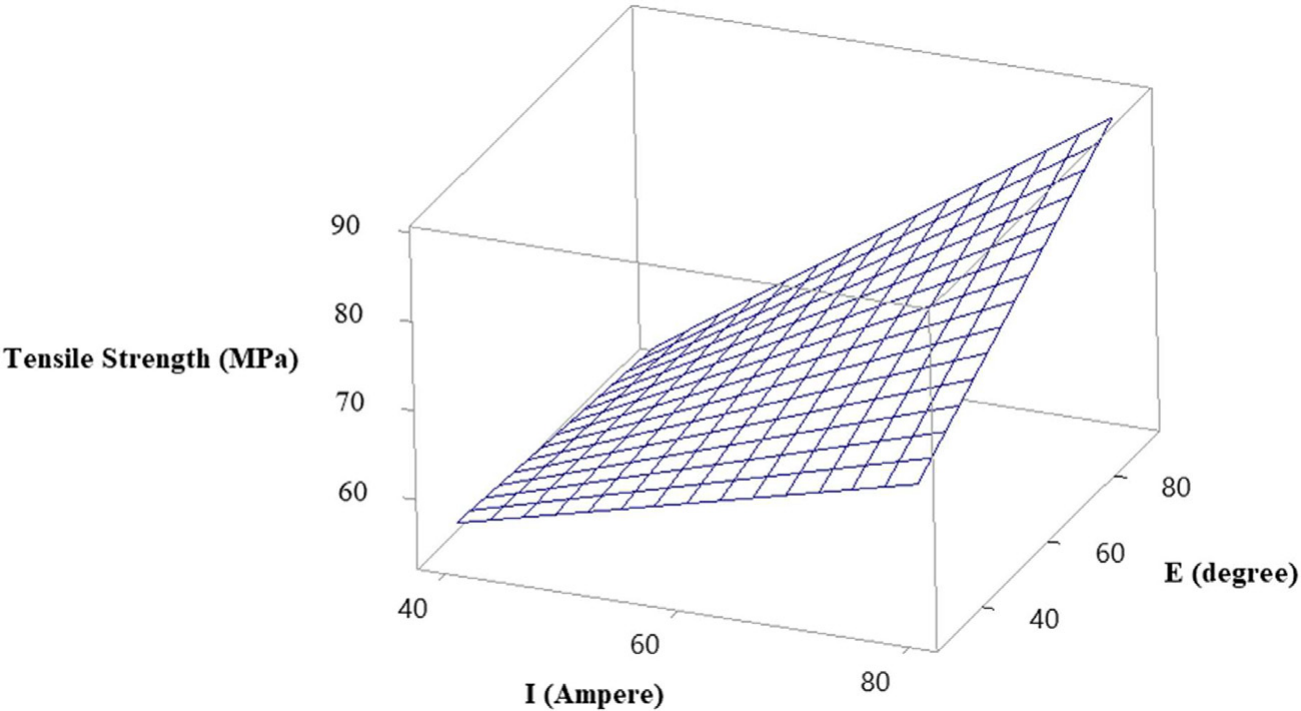
Surface Plot of Tensile Strength Vs Welding Current and Electrode Position.

**Fig. 4:**
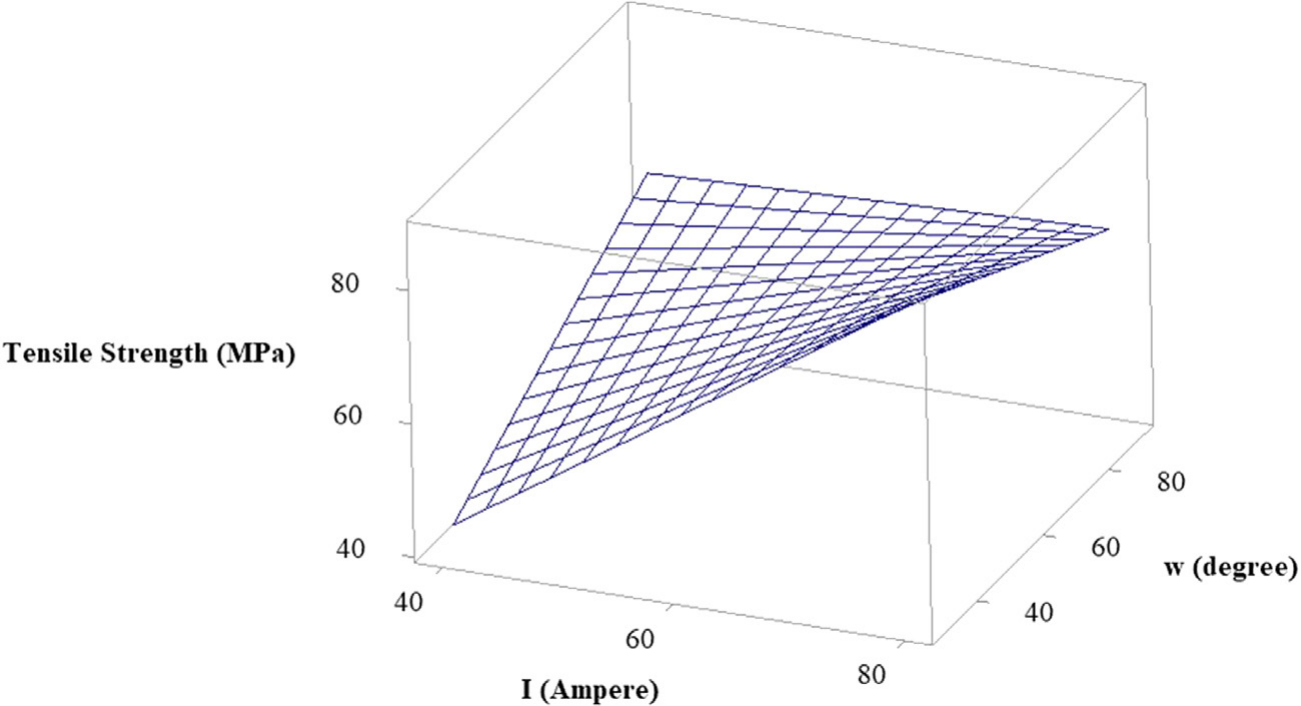
Surface Plot of Tensile Strength Vs Welding Current and Weld Orientation.

**Fig. 5:**
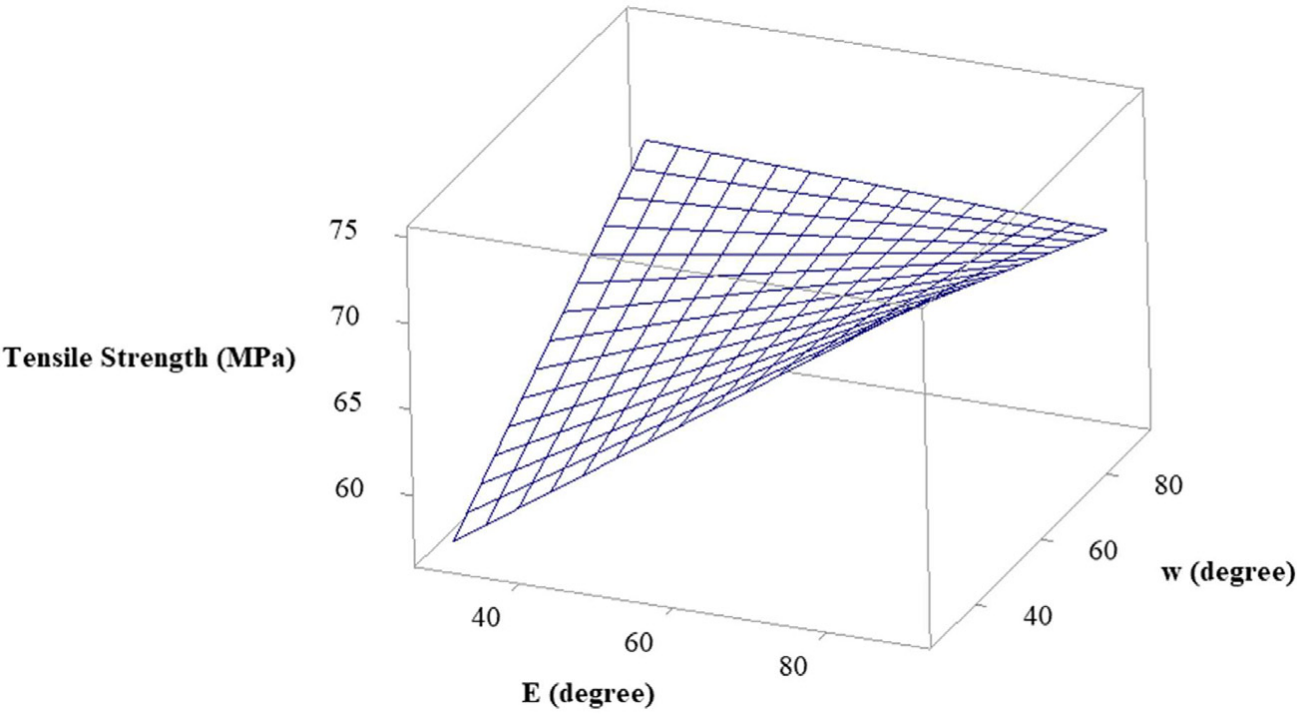
Surface Plot of Tensile Strength Vs Electrode Position and Weld Orientation.

Using the surface plots, it is viable to identify the optimal and critical regions within the range of the selected parameters. These regions are often characterized by high or low values of the output response. By examining the surface plot, we can locate regions where the response is maximized or minimized, providing insights into the optimal settings of the parameters. The abrupt changes in the surface plot indicate high sensitivity, while gradual changes suggest lower sensitivity. This information can be valuable in understanding the magnitude and rate of response changes concerning the variations in the MMAW parameters. The surface plots of tensile strength versus welding current and electrode position, tensile strength versus welding current and weld orientation, and tensile strength versus electrode position and weld orientation are depicted in Fig. 3, Fig. 4, Fig. 5 respectively.

In the statistical analysis, the contour plots of the MMAW parameters on the tensile strength provide insights into the relationship between the predictors and the response variable [11]. These plots visualize how the output response changes as the parameters vary and allow for inference and interpretation of the relationship. The contour plots can reveal interaction effects between the selected MMAW parameters. An interaction occurs when the effect of one parameter on the response depends on the level of another parameter. By examining the contour lines, we can observe if they are parallel or intersecting, thus indicating the presence or absence of interaction effects.

**Fig. 6:**
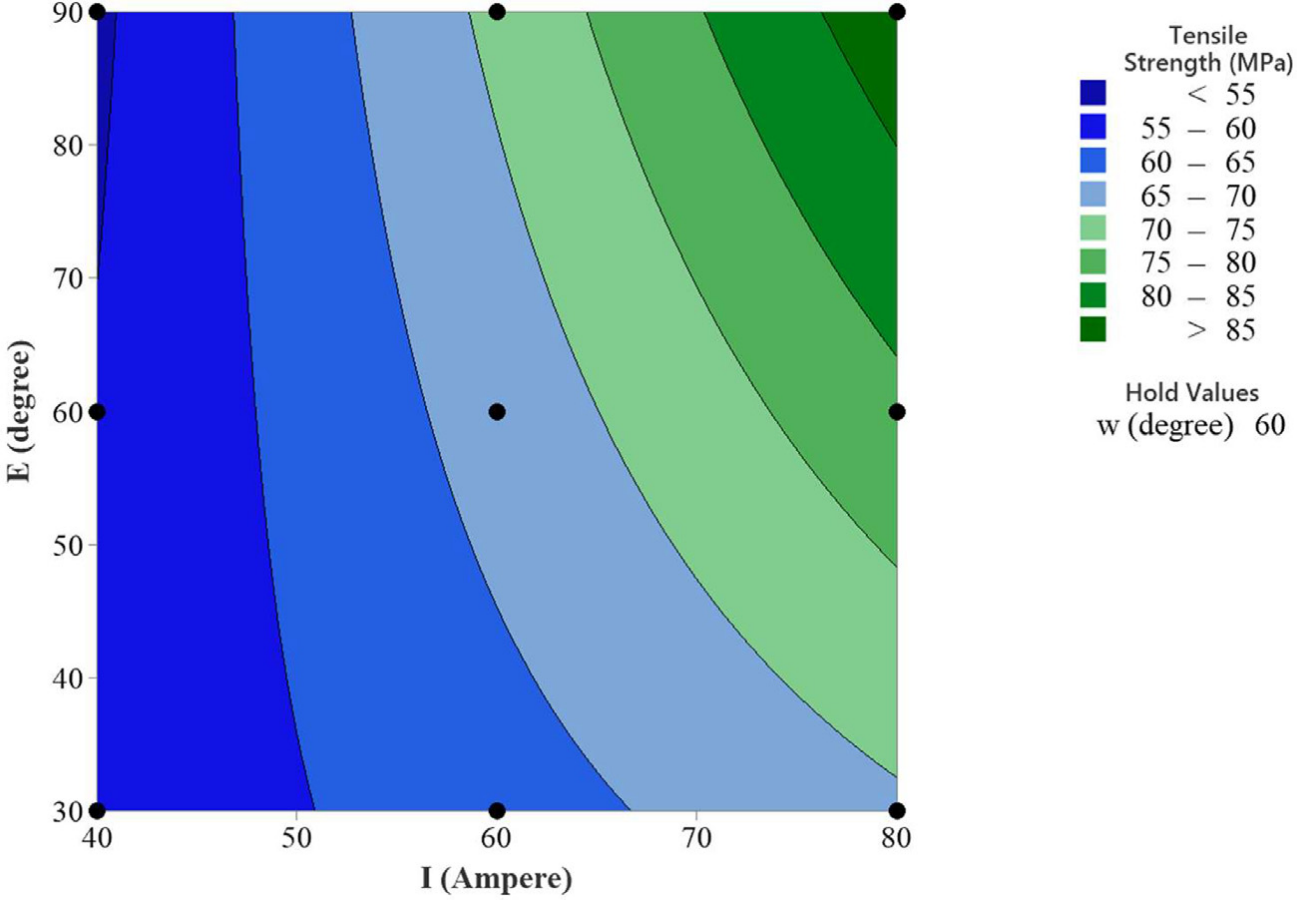
Contour Plot of Tensile Strength Vs Welding Current and Electrode Position.

**Fig. 7:**
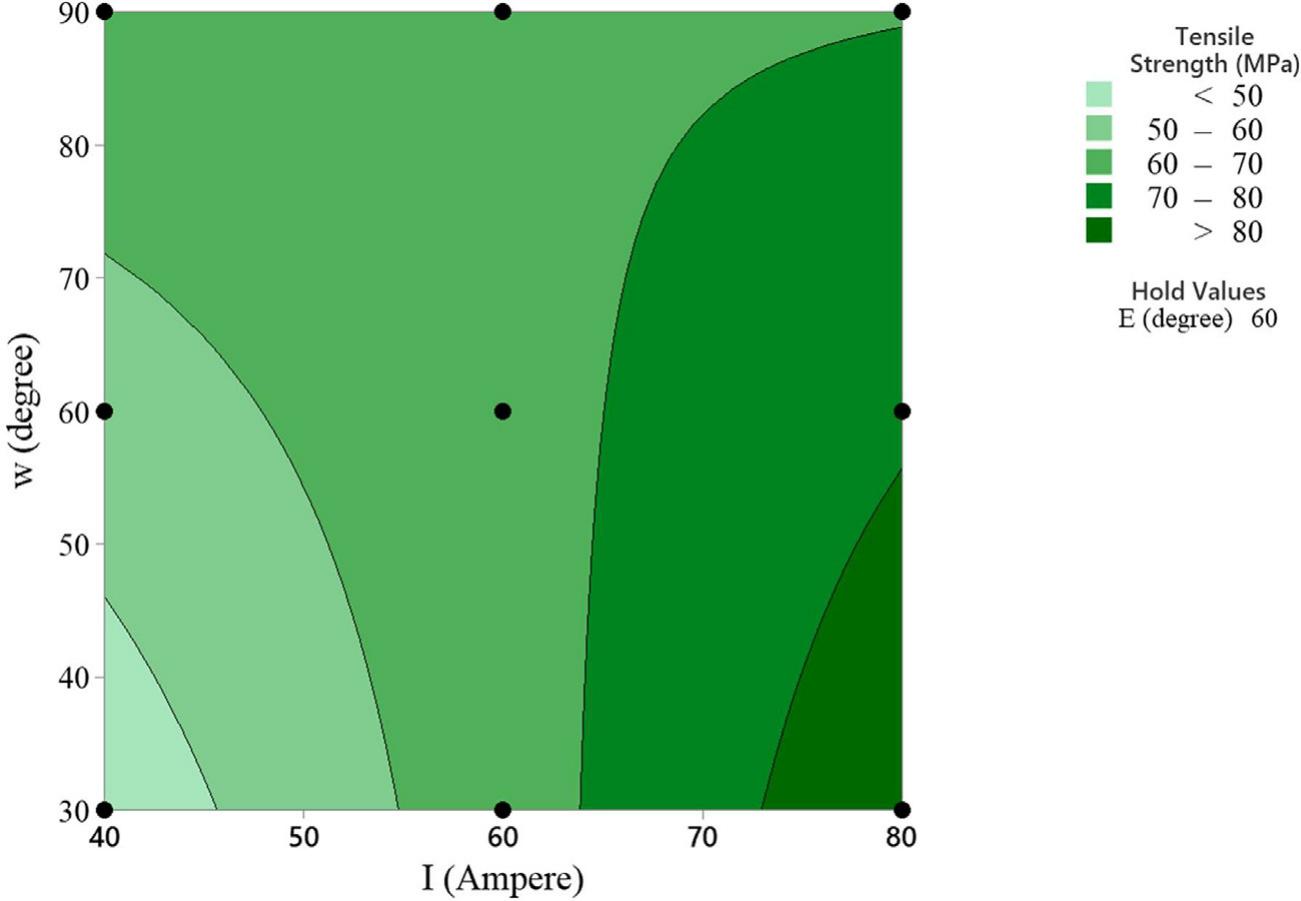
Contour Plot of Tensile Strength Vs Welding Current and Weld Orientation.

**Fig. 8:**
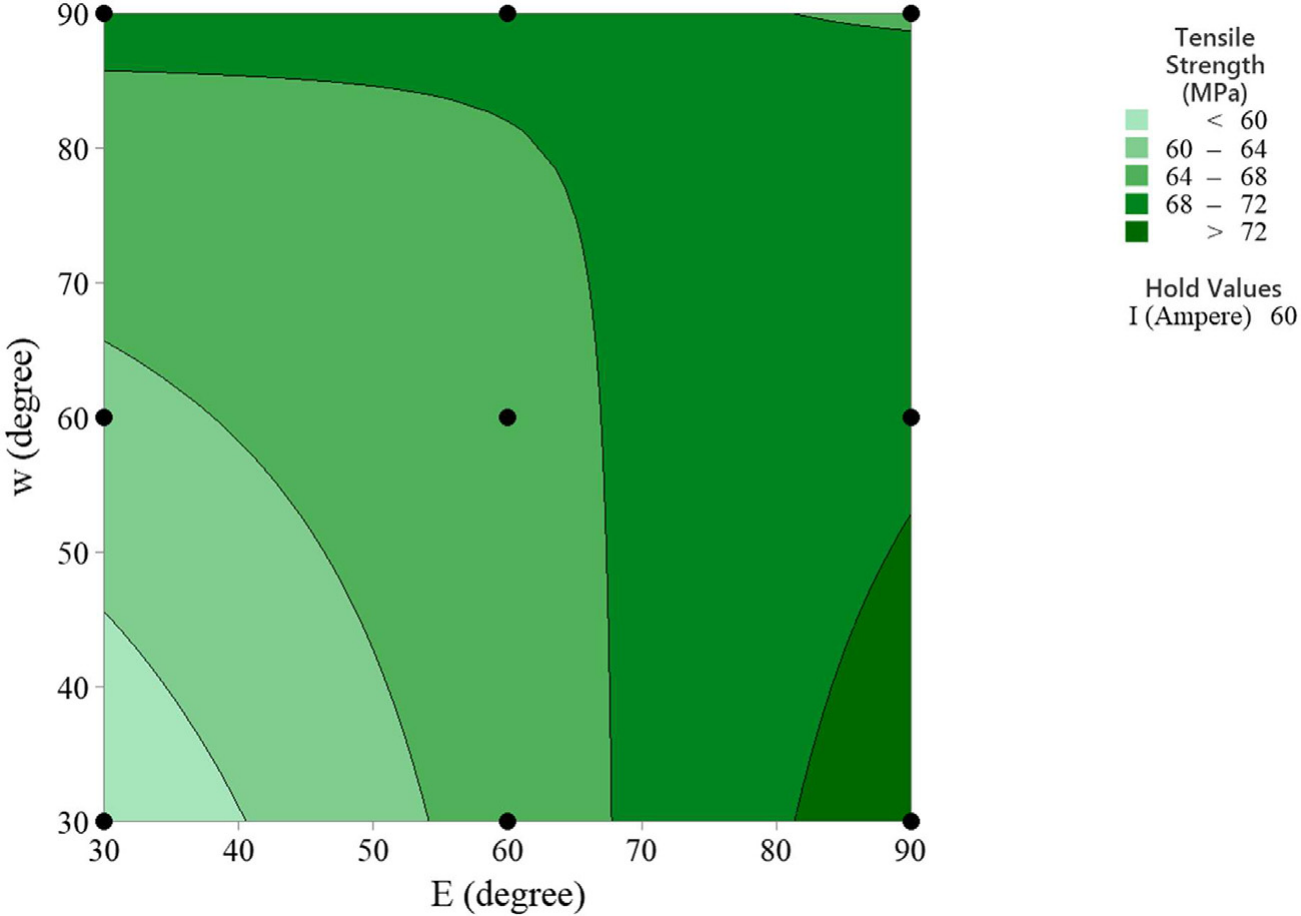
Contour Plot of Tensile Strength Vs Electrode Position and Weld Orientation.

The contour plots can also reveal the nonlinear relationships between the MMAW parameters and the response. If the contour lines are not parallel and exhibit irregular shapes, it suggests a nonlinear relationship. The contour plots of tensile strength versus welding current and electrode position, tensile strength versus welding current and weld orientation, tensile strength versus electrode position and weld orientation are depicted in Fig. 6, Fig. 7, Fig. 8 respectively.

**Fig. 9:**
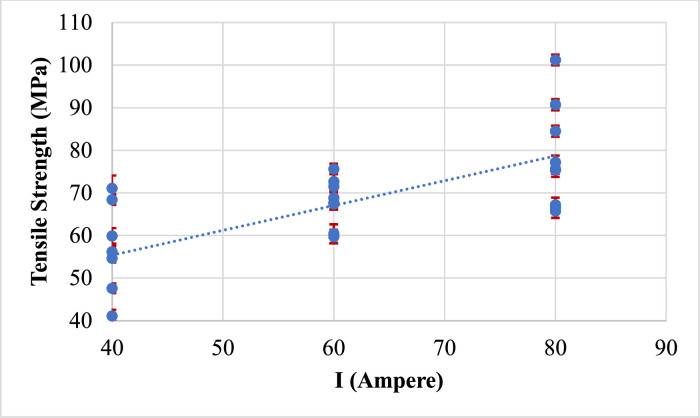
Effect of welding current on tensile strength.

**Fig. 10:**
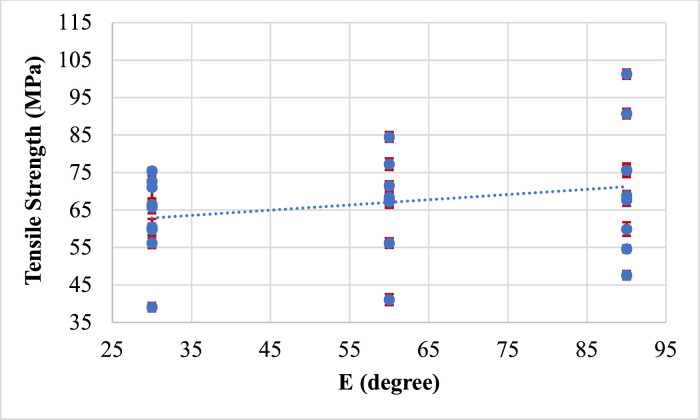
Effect of Electrode Position on tensile strength.

**Fig. 11:**
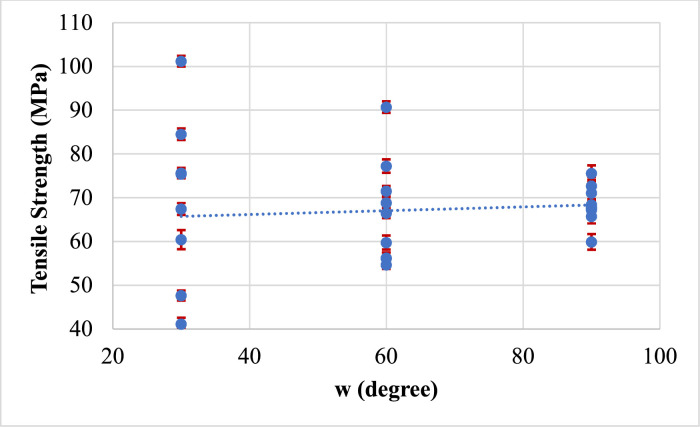
Effect of Weld Orientation on tensile strength.

A plot of the major response output tensile strength versus the MMAW parameters such as welding current, electrode position, and weld orientation are shown in Fig. 9, Fig. 10, Fig. 11 respectively.

**Fig. 12:**
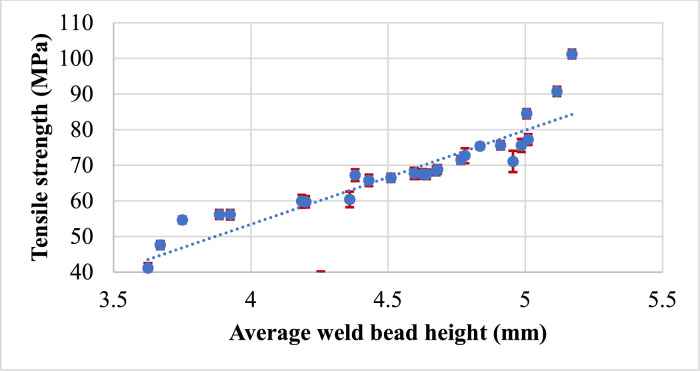
Tensile strength vs weld bead height.

**Fig. 13:**
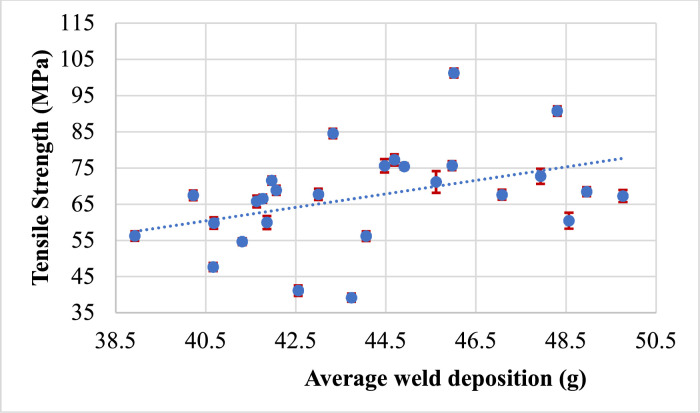
Tensile Strength vs Weld deposition.

Tensile strength is an important mechanical property of materials, and it can have a significant effect on weld bead height and weld deposition. The relationship between tensile strength Vs weld bead height and tensile strength Vs weld deposition can be complex and depends on various factors, including the material being welded, the welding process used, and the welding parameters. So, in this research work, we have compared the weld bead height and weld deposition with the tensile strength of the welded specimen for each trial. The effect of the supplementary responses such as weld bead geometry (height) and weld deposition on the major response output tensile strength are shown in Figs. 12 and 13 respectively.

## Limitations

Not Applicable.

## Ethics Statement

The authors did not conduct human or animal studies.

## CRediT authorship contribution statement

**Titus Wanazusi:** Methodology, Data curation, Software, Investigation, Writing – original draft. **Milon Selvam Dennison:** Conceptualization, Writing – original draft, Supervision, Validation, Writing – review & editing. **Stephen Ndubuisi Nnamchi:** Supervision, Writing – review & editing.

## Data Availability

Data Set of Manual Metal Arc Welded heterogeneous thin steel plates AISI1018 AND AISI4340 (Original data) (Mendeley Data)
